# Pediatric Interventions in a Sanfilippo Syndrome Patient Under General Anesthesia: A Case Report

**DOI:** 10.1155/crid/7892363

**Published:** 2025-01-06

**Authors:** Ahmad Al Malak, Hassan Issawi, Mohammad Hassoun, Mohammad Al Halabi

**Affiliations:** ^1^Faculty of Dental Sciences, Lebanese University, Beirut, Lebanon; ^2^Faculty of Dental Sciences, Beirut Arab University, Beirut, Lebanon; ^3^Department of Pediatric Dentistry, Faculty of Dental Medicine, Universite Saint-Joseph de Beyrouth, Beirut, Lebanon

## Abstract

**Background:** Mucopolysaccharidosis (MPS) Type III (MPS III) or Sanfilippo syndrome is a rare autosomal recessive inherited metabolic disorder. This disorder is responsible for lysosomal storage disorder at the cellular aspect. Due to lysosomal enzyme perturbance leading to the alteration of macromolecule metabolisms, this cellular perturbance causes multiple severe systemic and mental outcomes. Sanfilippo syndrome is the most frequent lysosomal disorder among the different types of MPS.

**Case Presentation:** A 9-year-9-month-old female was presented at our private clinic accompanied with her parents and referred from a general practitioner, and the preclinical examination revealed atypical craniofacial structure and skeletal features such as abnormal posture and movements adding on atypical behavioral manifestations such as temper tantrums, speech difficulties, dementia, and destructive behavior.

**Summary**

• Mucopolysaccharidosis (MPS) or Sanfilippo syndrome (mucopolysaccharidosis Type III (MPS III)) is a rare autosomal recessive inherited metabolic disorder.
• This disorder is responsible for lysosomal storage disorder at the cellular aspect.
• Due to lysosomal enzyme perturbance leading to the alteration of macromolecule metabolisms, this cellular perturbance causes multiple severe systemic and mental outcomes.
• MPS III is the most frequent lysosomal disorder among the different types of MPS; however, a small number of studies encountered the oral and dental manifestations of MPS III.
• This case report describes the oral and dental manifestations of a 9-year-old patient suffering from Sanfilippo syndrome and the appropriate treatment plan, which was conducted under general anesthesia.
• Our outcomes showed a crucial indication regarding undertaking complex or multiple dental procedures under general anesthesia for Sanfilippo patients, which was accompanied by no significant postoperative complications.

## 1. Introduction

Mucopolysaccharidosis (MPS) or Sanfilippo syndrome (mucopolysaccharidosis Type III (MPS III)) (Online Mendelian Inheritance in Man (OMIM) number: 252920) is a rare autosomal recessive inherited metabolic disorder. This disorder is responsible for lysosomal storage disorder at the cellular aspect. Due to lysosomal enzyme perturbance leading to the alteration of macromolecule metabolisms, this cellular perturbance causes multiple severe systemic and mental outcomes [1]. MPS III is the most frequent lysosomal disorder among the different types of MPS [2] since described for the first time in 1963 [3]. Epidemiologically, a range of 0.28–4.1 1 in 100,000 live births [4] suffers from MPS III with varying prevalence across geographic areas and predominant subtypes in specific regions of the world [5].

Alterations in lysosomal enzymes result in deficiency of glycosaminoglycan (GAG) heparan sulfate metabolism and the accumulation of GAG in the cell, followed by cellular damage that will affect several organ systems and failure of proper functioning of organs, leading to cognitive decline and progressive neurocognitive degeneration [1].

Symptoms typically emerge after a phase of normal growth and include speech delay, developmental dysfunctions, severe behavioral problems, and hyperactivity. Children affected by this syndrome also present a delay in language development or poor coordination with other children, along with some typical facial dimorphic features and dermatological manifestations such as congenital dermal melanocytosis. Moreover, with progressive cognitive decline, the patients eventually regress to a fully bedridden and vegetative state, which dramatically reduces their life expectancy [1, 6].

Regarding gastrointestinal outcomes, according to Thomas et al. [7], MPS patients suffer from gastrointestinal problems such as constipation, diarrhea, intolerance, and malabsorption of nutrition due to swallowing problems. In addition, patients above 10 years suffering from MPS III showed progressive cognitive processes and motor function loss, including swallowing and walking difficulties leading to gastrostomy feeding [7]. Moreover, regarding oral and dental outcomes, a wide range of anomalies can be described regarding the oral and dental outcomes, such as temporomandibular joint pathology, malocclusion, and high risk of caries mainly due to lack of hygiene due to movement and cognitive difficulties [8–10].

## 2. Medical History

In November 2023, a nine-year-nine-month-old female was presented at our private clinic accompanied by parents, referred from a general practitioner. The preclinical examination revealed atypical craniofacial structure and skeletal features such as abnormal posture and movements, adding on an atypical behavioral manifestation such as unpleasant and disruptive behaviors, emotional outbursts, speech difficulties, dementia, and destructive behavior and continuous carrying and pressing on the left cheek. Thus, based on the previous presentation, an atypical syndrome was suggested in order to accomplish a proper diagnosis. When she was 9 months old, the patient showed an extended crawling period, a hypoactive behavioral phase, a lack of communication and interaction with her environment, and a recurrent ear infection. After 5 months at 1-year-two-months-old, the patient was referred to the Department of Pediatrics and Adolescent Medicine at the American University of Beirut Medical Center. The Director of the Inherited Metabolic Diseases Program, based on the patient's symptoms, diagnosed the patient with mucopolysaccharidosis Type IIIA (MPS IIIA) (Sanfilippo syndrome). Additionally, GAG level in urine was assessed using a two-dimensional electrophoresis technique, revealing a high level of GAG excretion of 26 mg/mmol creatinine. However, no further information was presented regarding the genetic and additional biochemical tests. Regarding the current systemic status, the patient is introduced with no cardiovascular, kidney, or ophthalmic-related diseases, although systemic manifestations could accrue with age. In addition, the patient is not under any medications. Moreover, at 8-year-old, a regression pattern was observed regarding the musculoskeletal status until she became paraplegic; additionally, the neurocognitive state was assessed using the Sanfilippo Behavior Rating Scale [11]; however, no further cognitive tests were presented due to the socioeconomic status of the parents. According to the parents, no complications were observed during pregnancy and a normal virginal delivery. Although the patient was the second case of MPS in her family, this can shed light on the potential purpose of this genetic mutation, which is that the parents are first-degree related.

## 3. Oral Examination and Treatment Plan

**Figure 1:**
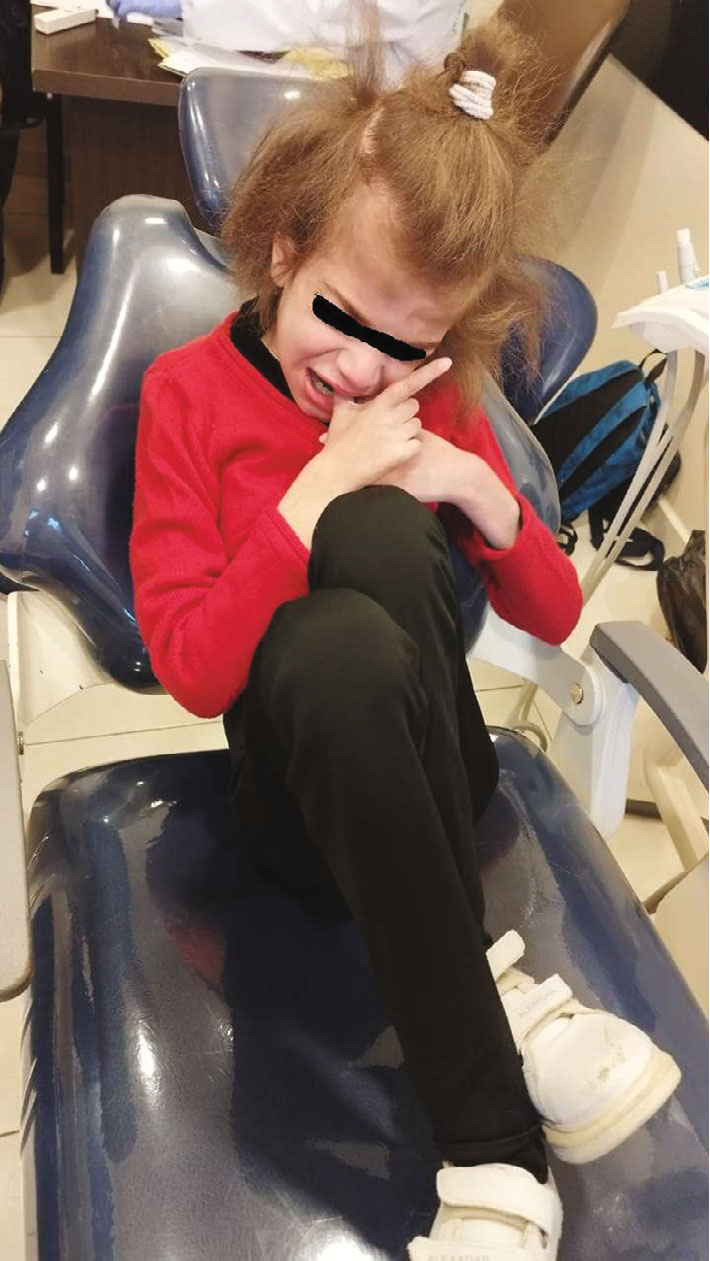
Mouth sucking habit conducted by the patient.

**Figure 2:**
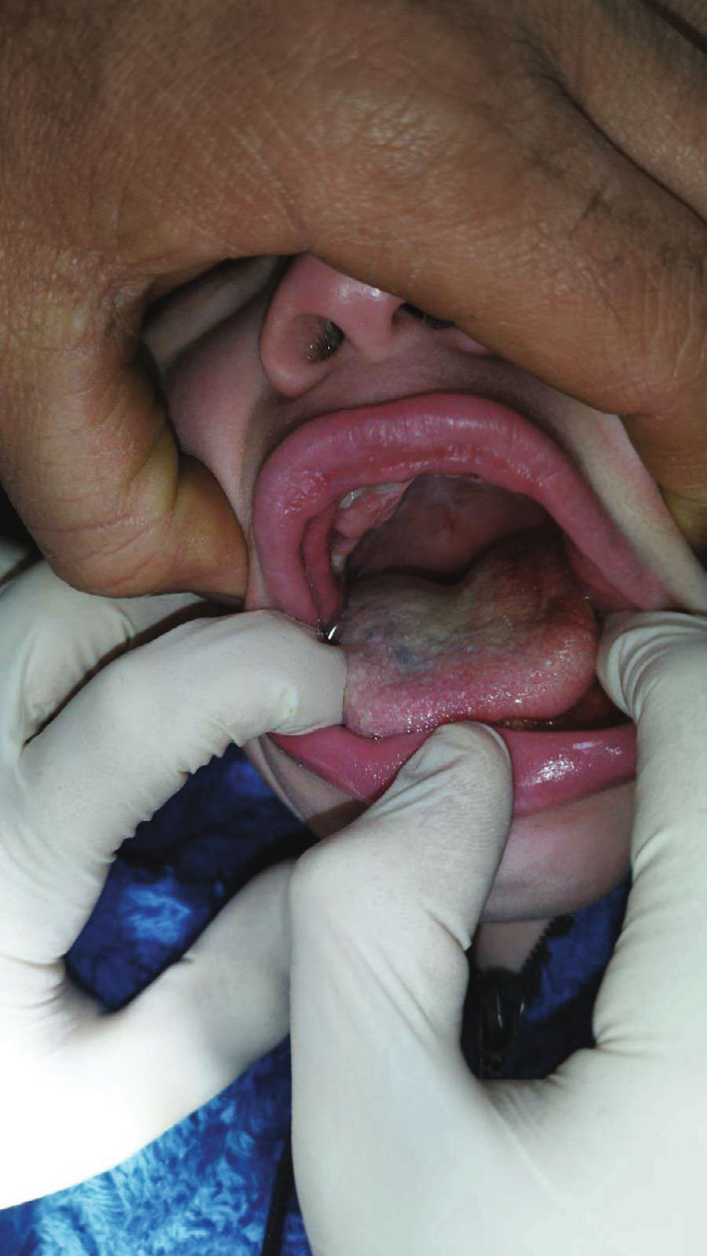
Patient presenting with a coated tongue.

**Figure 3:**
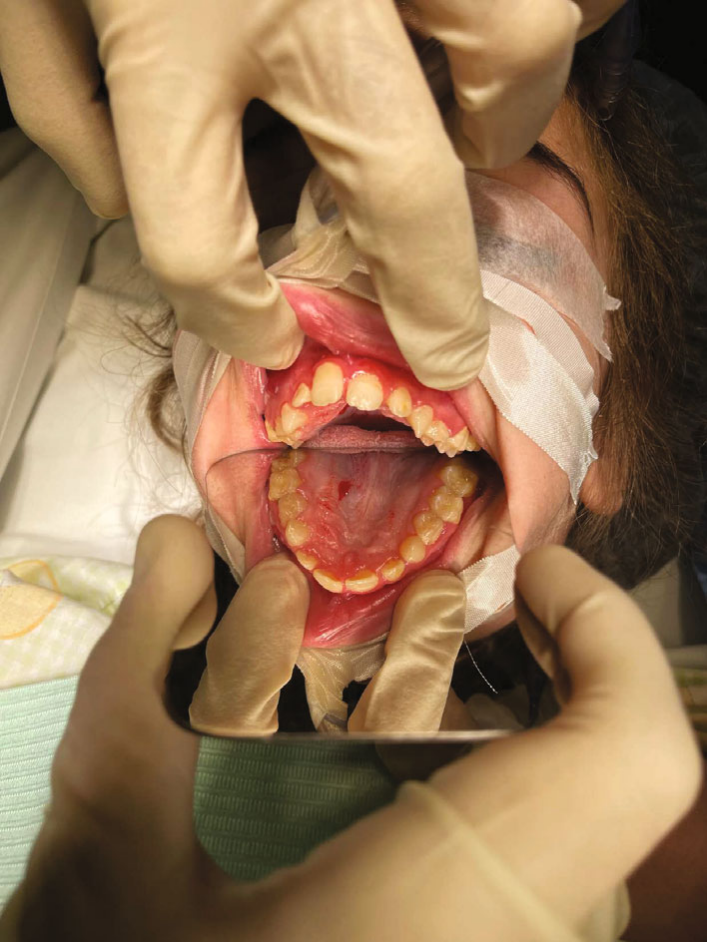
Patient presenting with a high-arched palate.

**Figure 4:**
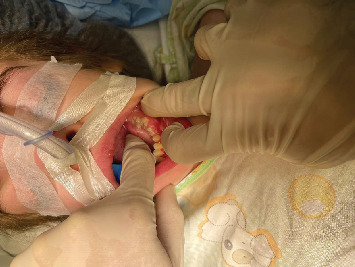
Generalized tartar and plaque in the molar region.

Before commencing any dental treatment in patients presenting MPS, an early diagnosis should be well discussed and consented to by any patient's guardian, clarifying any possible complications, especially in challenging cases. Based on the previous diagnosis, all precautions, such as proper posture and ergonomics, were adapted to the patient's disabilities, and the tell, show, do technique was conducted to relieve the anxious state of the patient. Concerning extraoral examination, an open bite was observed due to the thumb-sucking habit that was seen on the patient's first visit (Figure 1). Regarding intraoral examination, the patient had the typical oral features of a patient with MPS III, such as broad oral ridges and macroglossia and a coated tongue due to inadequate oral hygiene (Figure 2), hyperplasic gingiva, peg-shaped teeth, and a high-arched palate (Figure 3), and a generalized marginal gingival inflammation (generalized gingivitis) due to the presence of dispersed plaque and calculus mainly in the lingual anteroinferior region and upper first molar region (Figure 4) and inadequate oral hygiene. The gingiva was inflamed due to calculus visible on the upper first molars and lower incisors. Using the International Organization for Standardization (ISO) 3950 (World Dental Federation (FDI)) notation and the International Caries Detection and Assessment System (ICDAS), the intraoral examination revealed the following.

**Figure 5:**
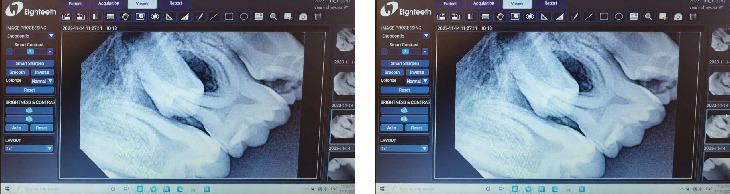
(a) Resorbed distal root due to the distal inclination of the erupting Premolar #35. (b) Resorbed distal root due to the distal inclination of the erupting Premolar #45.

The following treatment plan was suggested to be undergone: composite resin restorations on Tooth Number 16; in addition, based on the radiological findings of Munoz-Sanchez et al., and in order to obtain a stable chewing surface (Bourdiol P et al.), stainless steel crowns were placed on Tooth Numbers 26 and 46; moreover, simple extraction of Tooth Numbers 36, 54, 55, 64, 65, 74, 75, 85, and 84, and varnish application on Tooth Number 21 (Table 1 and Figure 5).

## 4. Anesthesia Management

The vast majority of dental procedures demand local or regional anesthesia, as it ensures a more comfortable and manageable treatment. However, any systemic dysfunctions could recommend applying generalized anesthesia. Patients suffering from MPS are considered quite challenging and could ask for an experienced anesthesiologist due to the gradual buildup of lysosomal GAGs, which is reflected in the poorly mental and physical status of the patient. MPS patients are considered challenging cases within the dental chair as they may have difficulty cooperating due to anxiety and behavioral changes. Also, depending on the severity of the disease, MPS patients could have an increased risk of supernumerary teeth, conoid teeth, taurodontism, impacted teeth, and root dilaceration. The presence of bone rarefaction/furcation lesions, condylar hypoplasia, and radiolucent bone lesions is also more frequent among MPS individuals [12]. Despite the dental abnormality, other systemic problems can assert that MPS patients are considered hard to deal with in comparison with normal, healthy patients [13]. Special considerations according to the different systemic involvements should be taken. As for monitoring the risk of mortality and morbidity, airway management was highly recommended [14]. A restricted nasal airway, macroglossia, adenotonsillar hypertrophy, a short, immobile neck, a thickened hyperglottis, or a generalized thickening of the brachioradial tree are all manifestations of specific airway involvement. Obstructive sleep apnea is reported to develop in up to 90% of children with MPS, as evidenced by positive polysomnography results [15]. According to Clark et al., MPS III showed no specific facial or airway characteristics, which may lead to general anesthesia complications [14]. However, Lao et al. asserted that their recent research revealed a lower rate of anesthesia complications than earlier studies [14, 16]. Moreover, regarding muscle weakness in MPS, which may lead to severe anesthetic complications, the anesthesiologist declared no significant risk regarding the usage of precautions related to muscle weakness. After obtaining written consent from the parents in late November 2023, a patient was presented at Haykel Hospital, where an intravenous line of insertion of propofol (40 mg), dexamethasone (4 mg), and rocuronium bromide (12 mg) was administered due to antinausea purposes. In addition, oral airway was used, and no complications were observed during manual ventilation. After induction, an easy nasotracheal intubation with a normal tube size of six and eye protection was achieved (Figure 4). In addition, the maintenance of general anesthesia was undergone only via inhalation of 3% sevoflurane. During the dental procedures, the heart rate and oxygen saturation were in the normal range (85–125 and 98%–99%, respectively); in addition, no sedation monitors were used. Moreover, no complications were noticed after the emergence phase, and the patient was discharged the same day. In addition, amoxicillin (1 g) and acetaminophen (300 mg), dexketoprofen (15 mg), and Tramadol (40 mg) were prescribed for postoperative antibiotic and pain management, respectively.

## 5. Discussion

According to Yoon et al., MPS patients, including those with MPS III, are at high risk of dental and oral diseases. GAGs build up in different tissues, such as teeth and gums, making people with MPS more susceptible to dental diseases, as this accumulation can interfere with normal tooth formation, leading to dental anomalies, elevated risk of cavities, and periodontal diseases [10, 17]. Depending on the severity of the disease, systemic manifestations could be observed, such as skeletal dysplasia, limited joint movement, and respiratory infections due to the excess of GAGs accumulated that can weaken the body's ability to fight infection, which in severe cases can lead to death [18].

**Figure 6:**
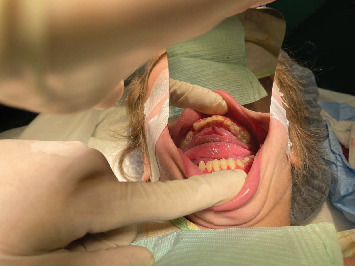
Macroglossia presented by the patient.

In addition, limited data is currently present describing oral features regarding MPS III in comparison with other forms of MPS; nevertheless, the oral findings of MPS III patients could be described as peg-shaped teeth, macroglossia (Figure 6), malocclusions, delayed tooth eruptions, and hyperplastic gingival (Figure 3).

**Figure 7:**
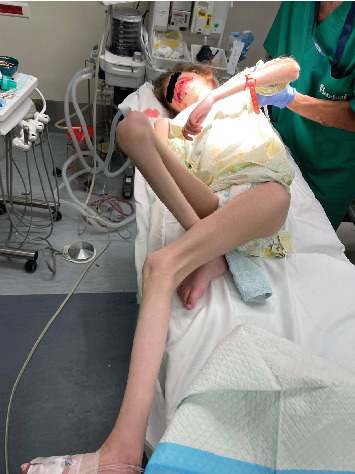
Advanced musculoskeletal symptoms in a Sanfilippo patient.

No doubt any dental procedure cannot perfectly restore a natural tooth; thus, prevention is always better than cure, especially when it comes to patients with MPS, as they face some behavioral difficulties and a higher risk of dental diseases. In addition, when dealing with disabled patients, guardians' follow-up is crucial. Lack of awareness is widely spread where parents only take their children for a dental checkup when they experience heavy pain [19], especially in Lebanon. However, in the United Kingdom, a bare minimum of awareness is achieved [19]. According to Muschol et al., patients should be motivated to brush their teeth twice a day in order to maintain good oral hygiene and to avoid sugary alimentation; moreover, in highly uncooperative patients (Figure 7), alternative approaches could be indicated, such as drinking water after sugary alimentation, wiping teeth regularly, using bite blocks, and three-sided toothbrushes [5].

As with any dental procedure, constant monitoring is a key to a successful treatment. In our case, if ongoing surveillance was neglected, a progressive resorption of the condyle could be remarkable, which in return might provoke issues in the mandibular articulation resulting in an open bite and facial asymmetry. However, condylar hypoplasia can also hinder the dental treatment by limiting the mouth opening [20]. Based on Chouinard, Kaban, and Peacock, to ascertain the course of the deformities, evaluate the prognosis, and develop an appropriate treatment plan, it is crucial to carry out an early diagnostic of the temporomandibular joint abnormalities and to monitor any risk of dentigerous cyst development with age. [13, 21].

At the date of this paper, no published data clarifies a universal dental management for MPS III. Furthermore, in accordance with Sari et al., the choice of undergoing the previously mentioned dental treatment plan under general anesthesia is based on poor cooperation due to the mental and physical incapacity to remain on the dental chair with an open mouth [22]. In addition, based on several studies, no significant pre-, per-, or postoperation complications occurred after general anesthesia in MPS III [23–25].

**Figure 8:**
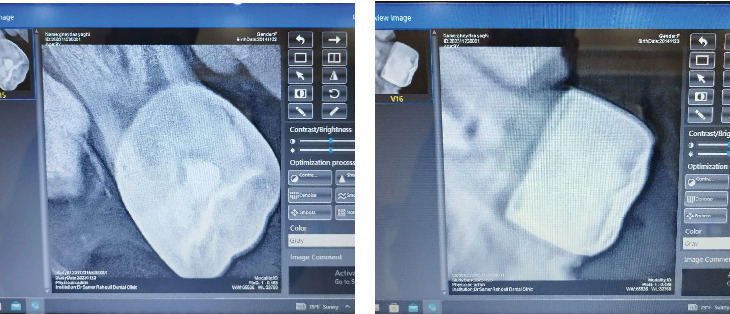
(a) Radiograph of the stainless steel crown placed on Tooth #26. (b) Radiograph of the stainless steel crown placed on Tooth #46.

Regarding dental interventions, stainless steel crowns were placed on two permanent teeth (Figures 8(a) and 8(b)). This procedure was supported by the radiographic findings of Munoz-Sanchez et al., showing no periodontal complications and a high success rate in special circumstances such as under general anesthesia [26]. In addition, a mouth opener was used along the procedures to obtain the mouth open (Figure 4).

This paper will serve as an invitation for the upcoming studies targeting MPS III patients in the Arab world and the Middle East, as it is the first case report on MPS III patients in the region. In addition, our paper showed no complications were present when undergoing the general anesthesia, contrary to some other studies [14, 27] since all precautions were seriously taken into consideration.

## Figures and Tables

**Table 1:** Results of the intraoral examination.

**Quadrant 1**	**Quadrant 2**
Tooth #16: Vital with occlusal carious lesion Type 3 ICDAS	Tooth #21: A hypomineralization spot on its labial surface (Figure 3)
Tooth #54: In a loose state and close to its exfoliation time	Tooth #64: In a loose state and close to its exfoliation time
Tooth #53: In a loose state and close to its exfoliation time	Tooth #63: In a loose state
**Quadrant 4**	**Quadrant 3**
Tooth #84: In a loose state and close to its exfoliation time	#36: Necrotic with apical periodontitis, carious lesion Type 6 ICDAS
Tooth #85: Occlusal carious lesion Type 4 ICDAS and resorbed distal root due to distal inclination of the erupting Premolar #45 (Figure 5(b))	#75: Occlusal carious lesion Type 4 ICDAS and resorbed distal root due to distal inclination of the erupting Premolar #35 (Figure 5(a))
Tooth #46: Vital with occlusal carious lesion Type 5 ICDAS	Tooth #74: In a loose state and close to its exfoliation time

## Data Availability

All data are included in this manuscript.
